# The narrow window of protection: protective efficacy of maternally derived antibodies against virulent classical swine fever virus in Japan

**DOI:** 10.1186/s13567-025-01583-z

**Published:** 2025-07-16

**Authors:** Katsuhiko Fukai, Tatsuya Nishi, Mitsutaka Ikezawa, Rie Kawaguchi, Kazuki Morioka

**Affiliations:** WOAH Reference Laboratory for Classical Swine Fever, Kodaira Research Station, National Institute of Animal Health, National Agriculture and Food Research Organization, 6-20-1 Josui-Honcho, Kodaira, Tokyo 187-0022 Japan

**Keywords:** Classical swine fever, clinical manifestation, experimental infection, infection, maternal antibody, protective threshold

## Abstract

This study investigated the protective efficacy of maternally derived antibodies (MDAs) against the currently circulating virulent classical swine fever virus (CSFV) strain in Japan (JPN/1/2018). Thirty-eight piglets from guinea-pig exaltation of Newcastle disease virus-negative phenomenon (GPE^−^)–vaccinated (Groups 1–2) or unvaccinated sows (Group 3) were divided into three groups: Group 1 (*n* = 16, MDA titres < 2 to 5.6 against JPN/1/2018), Group 2 (*n* = 16, MDA titres 45–362), and Group 3 (*n* = 6, no MDAs). All piglets were orally challenged with the JPN/1/2018 strain and monitored for clinical signs, viremia, viral shedding, and viral tissue distribution. Groups 1 and 3 showed similar clinical manifestations, viremia, viral shedding and viral distribution patterns, whereas Group 2 piglets generally remained clinically normal with limited viral detection in clinical samples and organs. On the basis of these results, an MDA titre of 45 against JPN/1/2018 was determined as the approximate protective threshold. The use of antigenic relatedness (r_1_) values between the JPN/1/2018 and GPE^−^ strains (mean r_1_ = 0.43) corresponds to an MDA titre of approximately 105 against the GPE^−^ strain. However, two Group 2 piglets whose MDA titres were above the threshold died during the experiment, suggesting that individual factors such as body size, social hierarchy, and health status may influence protection. The narrow window between the protective threshold (MDA titre against the GPE^−^ strain of 105) and the titre that interferes with the vaccine-induced antibody response (MDA titre against the GPE^−^ strain of ≥ 128) presents challenges for farm management, highlighting the importance of combining vaccination with strict biosecurity measures.

**Supplementary Information:**

The online version contains supplementary material available at 10.1186/s13567-025-01583-z.

## Introduction

Classical swine fever virus (CSFV) is a member of the genus *Pestivirus* within the family *Flaviviridae* and possesses a positive single-stranded RNA genome of approximately 12 300 nucleotides [1]. The virus is classified into three major distinct genotypes, each comprising several subgenotypes [1]. CSFV is a highly contagious, multisystemic, haemorrhagic disease in swine (*Sus scrofa*) that affects both domestic pigs and wild boars [1]. Infection with highly virulent CSFV strains can lead to high mortality rates and substantial economic losses. Furthermore, outbreak notifications typically trigger international trade restrictions, resulting in additional economic impacts.

In September 2018, a CSF outbreak occurred in Gifu Prefecture, Japan, the country’s first outbreak in 26 years [2]. As of March 2025, authorities have identified 96 cases on domestic pig farms across 23 prefectures [3]. Moreover, RT-PCR/real-time RT-PCR of blood and organ samples detected CSFV in more than 8300 wild boars across 39 prefectures during the same period [3]. In response, authorities initiated the routine administration of a bait vaccine (RIEMSER Schweinepestoralvakzine, Riemser Arzneimittel AG, Greifswald-Insel Riems, Germany) to wild boars in March 2019. In October, they subsequently began regular administration of a live attenuated guinea-pig exaltation of Newcastle disease virus phenomenon-negative (GPE^−^) vaccine to domestic pigs.

The transfer of maternally derived antibodies (MDAs) from immunized sows to piglets through lactation serves as an essential mechanism of passive immunity against viral infections [4, 5]. This protection is particularly crucial during the period before piglets can develop immunity through direct vaccination [4, 5]. In piglets that have ingested minimal colostrum, vaccination induces a strong antigenic stimulus, leading to a robust humoral immune response and resistance to subsequent viral challenges [6–8]. While several studies have examined whether piglets with passive antibodies obtained from colostrum can develop immunity following attenuated live CSF vaccination [6–9], few studies have evaluated the protective efficacy of MDAs alone against challenges with virulent virus strains [10–12].

This study aimed to determine whether piglets with varying levels of MDAs acquired from sows immunized with the GPE^−^ vaccine are protected against challenge with a virulent CSFV strain currently circulating in Japan.

## Materials and methods

### Facility

All experimental infections involving infectious viruses were conducted in 14 m^2^ cubicles within a high-containment facility at the National Institute of Animal Health, Japan. The cubicles were maintained at 25 °C with 10–15 air changes per hour throughout the study period. The high-containment facility meets the containment requirements for group 4 pathogens specified in the WOAH Manual of Diagnostic Tests and Vaccines for Terrestrial Animals, thirteenth edition 2024 [13].

### Cells and virus

CPK cells [14] were cultured in Dulbecco’s modified Eagle’s medium: nutrient mixture F12 (Thermo Fisher Scientific, Waltham, MA, USA) supplemented with 10% pestivirus-free fetal bovine serum. This study utilized the CSFV JPN/1/2018 strain [15], which was isolated from the first reported case in Japan using CPK cells and underwent two passages in the same cell line. Previous experimental infections confirmed that the JPN/1/2018 strain exhibited moderate virulence [16].

### Experimental infection

All the piglets were clinically healthy at the start of the experiment, showing no signs of illness or preexisting disease conditions. This study included thirty-two piglets aged 17–57 days with varying MDA titres against the JPN/1/2018 strain (ranging from < 2 to 362; Groups 1 and 2; *n* = 16 each) and six piglets aged 41–57 days without MDAs (Group 3) (Table 1). The selection of piglets with the observed MDA titre distribution was influenced by practical constraints in the experimental design. Since MDA titres can be definitively measured only from serum collected immediately before viral challenge and neutralization testing requires approximately 1 week to complete, we employed a predictive selection strategy. Candidate sows were selected on the basis of their neutralizing antibody titres measured 3–10 weeks before the expected farrowing date. Preliminary MDA testing was performed on candidate piglets from Group 1 at 29–36 days of age and from Group 2 at 3–5 days of age. These measured values, combined with an estimated MDA half-life of approximately 10 days, were used to predict the MDA titre at the time of challenge. Despite these efforts to obtain a continuous range of MDA titres, individual variation in MDA half-lives resulted in the absence of piglets with MDA titres between 5.6 and 45 at the time of challenge.

**Table 1:** **MDA titres of piglets in Groups 1 to 3**

Group	Pig #	MDA titre
1	1	5.6
	2	4
	3	4
	4	2
	5	2
	6	2
	7	2
	8	< 2
	9	< 2
	10	< 2
	11	< 2
	12	< 2
	13	< 2
	14	< 2
	15	< 2
	16	< 2
2	17	362
	18	362
	19	256
	20	256
	21	128
	22	128
	23	128
	24	90
	25	90
	26	90
	27	64
	28	64
	29	64
	30	64
	31	45
	32	45
3	33	< 2
	34	< 2
	35	< 2
	36	< 2
	37	< 2
	38	< 2

The Group 1 piglets originated from a conventional pig farm, whereas the Groups 2 and 3 piglets originated from different specific pathogen-free pig farms.

The Group 1 piglets, aged 50–57 days, were offspring of three sows and were subsequently mixed into a single group. Two of the three sows had received the GPE^−^ vaccine three times, while the remaining sows had received it twice. The neutralizing antibody titres of these sows against JPN/1/2018, measured 17 to 24 days before delivery, were 8, 64, and 64, respectively.

The Group 2 piglets, aged 17–28 days, were offspring of six sows and were subsequently mixed into a single group. All six sows had received the GPE^−^ vaccine twice. The neutralizing antibody titres of these sows against the JPN/1/2018 strain, which were measured 58 to 69 days before delivery, were 128, 181, 256, 256, 256, 362, and 362, respectively.

The Group 3 piglets, aged 41–57 days, were offspring of two sows that had not received the GPE^−^ vaccine and did not have neutralizing antibodies against the JPN/1/2018 strain before delivery. These piglets were subsequently mixed into a single group.

The age differences among groups were intentionally designed to achieve the target MDA titre levels for each group. Group 1 piglets (50–57 days old) were selected to obtain lower MDA titres through natural decay, whereas Group 2 piglets (17–28 days old) were selected to retain higher MDA titres. Group 3 served as a control group with MDA-negative piglets aged 41–57 days.

All piglets were orally inoculated with 2 mL of the JPN/1/2018 strain at a titre of 10^5^ 50% tissue culture infectious dose (TCID_50_), following previously described methods [17]. Throughout the infection period, serum, whole blood, and oral swabs were collected at 1–2 day intervals. Clinical scores were assessed using established criteria [18]. When clinical deterioration became severe, piglets were euthanized upon reaching a humane endpoint, defined as the inability to move or stand.

Necropsies were performed on all deceased animals and on surviving animals at the end of the experimental period. Organ samples from the brain, tonsil, spleen, kidney, adrenal gland, colon, and mesenteric lymph nodes were collected for immunohistochemical examination and viral detection as described above. Complete differential testing for other swine pathogens was not performed.

### RNA extraction and real-time reverse-transcription polymerase chain reaction

Viral RNA was extracted from clinical samples using the MagMAX CORE Nucleic Acid Purification Kit (Thermo Fisher Scientific) and the KingFisher Flex Purification System, KingFisher, with a 96 deep-wellhead (Thermo Fisher Scientific). Viral genes were detected using the CSFV/ASFV Direct RT‒qPCR Mix & Primer/Probe (with ROX Reference Dye) (Takara Bio, Shiga, Japan) [19].

### Viral titration

Viral titration was performed as previously described [20]. Briefly, clinical samples were subjected to tenfold serial dilutions and inoculated into four wells of 96-well plates containing CPK cells for each dilution. The cells were incubated at 37 °C with 5% CO_2_ for 7 days and then fixed with 80% acetone. Immunofluorescence staining was performed using an anti-CSFV E2 monoclonal antibody (WH303, Animal and Plant Health Agency, Surrey, UK) as the primary antibody and the goat anti-mouse IgG (H+L) cross-absorbed secondary antibody Alexa Fluor 488 (Thermo Fisher Scientific) as the secondary antibody. Viral antigens were visualized using an LSM700 microscope (Zeiss, Baden-Württemberg, Germany), and viral titres were calculated using the Reed and Müench method [21].

### Neutralization test

Antibody titres against the JPN/1/2018 and GPE^−^ strains were measured using a neutralization test as previously described [20]. Serum samples were mixed with an equal volume of chloroform to remove infectious CSFV and then centrifuged at 14 000 × *g* for 10 min at 4 °C. The supernatant was heated at 56 °C for 30 min to inactivate complement. The chloroform-treated and heat-inactivated sera underwent twofold serial dilutions and were mixed with 100 TCID_50_ of the JPN/1/2018 or GPE^−^ strains. After incubation at 37 °C for 1 h, the serum‒virus mixtures were added to CPK cell monolayers in 96-well plates and incubated at 37 °C with 5% CO_2_ for 7 days. Fixation and immunostaining procedures were performed as described for viral titration. The neutralizing antibody titres are expressed as the reciprocal of the highest serum dilution that inhibited virus growth in 50% of the duplicate wells.

To evaluate the serological relationship between JPN/1/2018 and GPE^−^ strains, an r_1_ value [22] was calculated using MDA titre at 0 days post-inoculation (dpi) in Group 2 piglets. These piglets were born to sows vaccinated with the GPE^−^ strain and had MDAs against this strain. The serum samples collected at 0 dpi contained only colostrum-derived MDAs against the GPE^−^ strain, as collection preceded JPN/1/2018 inoculation. Therefore, these samples, which function as antisera against the GPE^−^ strain, were suitable for evaluating the serological relationship between the two strains. The r_1_ value between the JPN/1/2018 and GPE^−^ strains was calculated using the following formula:

$${\text{r}}_{1} \;{\text{value}} = \frac{{{\text{MDA}}\;{\text{titer}}\;{\text{against}}\;{\text{JPN}}/1/2018\;{\text{strain}}}}{{{\text{MDA}}\;{\text{titer}}\;{\text{against}}\;{\text{GPE}}^{ - } \;{\text{strain}}}}$$

### Immunohistochemical examination

Organ samples collected from the brain, tonsil, spleen, kidney, adrenal gland, colon, and mesenteric lymph nodes were fixed in 10% neutral buffered formalin and embedded in paraffin wax using standard procedures. Deparaffinized organ sections were processed using the universal immunoenzyme polymer method with HISTONE Simple Stain Max PO (Nichirei, Tokyo, Japan) according to the manufacturer’s instructions. The antigen was detected with an anti-CSFV E2 monoclonal antibody, and the sections were counterstained with haematoxylin.

The immunohistochemistry results were scored using the immunoreactive score (IRS) system and classified into four categories: IRS 0–1 = negative (−), 2–3 = weakly positive (+), 4–8 = moderately positive (++), and 9–12 = strongly positive (+++) [23]. The IRS was calculated by multiplying the proportion of positive cells by the staining intensity. The proportion of positive cells was scored as follows: 0 = no positive cells, 1 = < 10%, 2 = 10–50%, 3 = 51–80%, and 4 =  ≥ 80%. The staining intensity was scored as follows: 0 = no staining, 1 = weak, 2 = moderate, and 3 = strong.

### Statistical analysis

Clinical scores, body temperature, and leukocyte counts were analysed using repeated-measures ANOVA with group and time as factors, followed by Tukey’s multiple comparison test for post hoc analysis. Survival analysis was performed using Kaplan‒Meier curves with the log-rank test. Viral gene detection rates, virus isolation rates, and viral antigen detection rates in different sample types and organs were compared using Fisher’s exact test. For the comparison of viral detection patterns between the high-MDA (≥ 128) and low-MDA (< 128) subgroups within Group 2, detection periods and days with Ct values < 30 were compared using the Mann‒Whitney U test, which is appropriate for nonparametric data with small sample sizes. A *p* value < 0.05 was considered to indicate statistical significance.

## Results

### Clinical manifestations and scores

**Figure 1:**
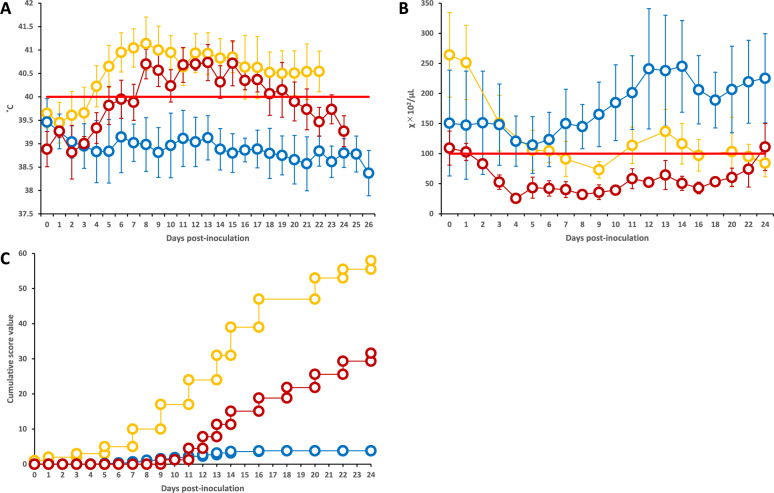
**Body temperature, leukocyte count and clinical score of the piglets in Groups 1–3.** **A** Body temperature (°C). **B** Leukocyte count (cells/µL). **C** clinical score. The results for Groups 1, 2, and 3 are represented by orange, blue, and red line graphs, respectively. Standard deviation values are also shown for body temperature and leukocyte count. In Groups 1 and 3, some piglets died during the experiment; therefore, the mean values after death were calculated using data from surviving piglets only.

Group 3 piglets developed fever (> 40 °C) beginning at 8 dpi and leukopenia (< 10 000 cells/µL) beginning at 2 dpi (Figure 1A and B). These animals exhibited multiple clinical signs, including depression, tremors, anorexia, diarrhea, ataxia, and erythema. The daily clinical score peaked at 32 (Figure 1C).

The patterns of Group 1 piglets were similar to those of Group 3 piglets, who developed fever (> 40 °C) from 4 dpi and leukopenia (< 10 000 cells/µL) from 7 dpi (Figure 1A and B). These animals displayed clinical signs similar to those in Group 3 and additionally exhibited coughing, conjunctivitis, and death. Three piglets died at 9, 18, and 22 dpi, with daily clinical scores reaching a maximum of 58 (Figure 1C).

Group 2 piglets generally remained clinically normal, showing neither fever (> 40 °C) nor leukopenia (< 10 000 cells/µL) (Figure 1A and B). However, two out of the 16 piglets in this group developed clinical signs, including diarrhea, anorexia, depression, ataxia, and emaciation, and died at 15 and 16 dpi. While surviving Group 2 piglets maintained low clinical scores (maximum of 4) (Figure 1C), the two affected piglets reached maximum scores of 20 and 31 before death (data not shown).

Compared with those of Groups 1 and 3, Group 2 piglets presented significantly lower clinical scores throughout the experimental period (repeated-measures ANOVA, *p* < 0.001). Group 2 maintained a normal body temperature (mean 38.7 °C) and leukocyte count (mean 21 000 cells/µL), whereas Groups 1 and 3 presented fever (40.7 °C and 40.3 °C, respectively) and leukopenia (10 150 and 4 960 cells/µL, respectively) (*p* < 0.001 for all comparisons vs Group 2).

Survival analysis revealed final survival rates of 81.3% (13/16), 87.5% (14/16), and 100% (6/6) for Groups 1, 2, and 3, respectively. Deaths occurred at days 9, 18, and 22 in Group 1 and at days 15 and 16 in Group 2. No deaths were observed in Group 3. Kaplan‒Meier survival analysis with the log-rank test revealed no statistically significant differences among the groups (*p* > 0.05).

### Detection of viral genes and viral titres

**Figure 2:**
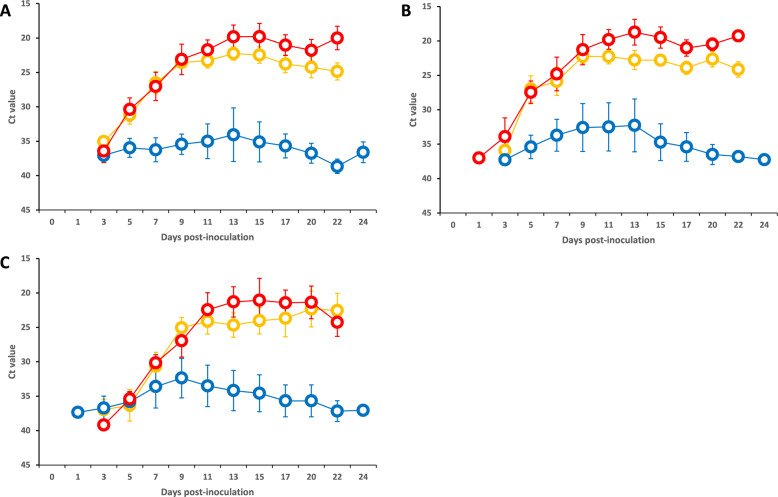
**RT-qPCR detection of viral genes in clinical samples from piglets in Groups 1–3.** **A** Serum. **B** Whole blood. **C** Oral swab. The results for Groups 1, 2, and 3 are represented by orange, blue, and red line graphs, respectively. The error bars indicate standard deviations. In Groups 1 and 3, some piglets died during the experiment; therefore, the mean values after death were calculated using data from surviving piglets only.

In Group 3, viral genes were initially detected in serum, whole blood, and oral swab samples at 3, 1, and 3 dpi, respectively (Figure 2, Additional file 1). The initial Ct values of the clinical samples were ≥ 30 for 1–4 days after the first detection and then gradually decreased to reach a minimum value of approximately 19. Viral genes were also detected in tonsil, spleen, kidney, adrenal gland, brain, colon, and mesenteric lymph node samples collected at necropsy, with Ct values ranging from approximately 18 to 32 (Additional file 2).

**Figure 3:**
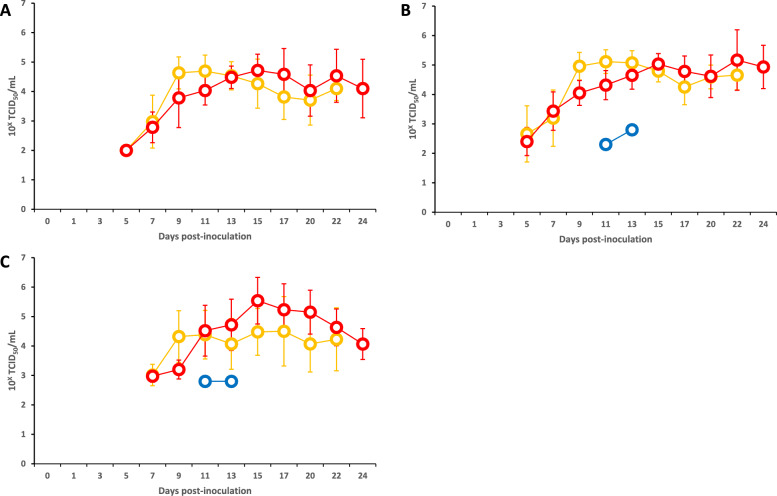
**Detection of infectious viruses in clinical samples from piglets in Groups 1–3.** **A** Serum. **B** Whole blood. **C** Oral swab. The results for Groups 1, 2, and 3 are represented by orange, blue, and red line graphs, respectively. The error bars indicate standard deviations. In Groups 1 and 3, some piglets died during the experiment; therefore, the mean values after death were calculated using data from surviving piglets only.

The infectious virus was initially isolated from serum, whole blood, and oral swab samples collected from Group 3 piglets at 5, 5, and 7 dpi, respectively (Figure 3, Additional file 3). The initial viral titres in the clinical samples ranged from 10^2.0^ to 10^3.4^ TCID_50_/mL for 2 days after the first isolation and then gradually increased to a peak at 10^5.5^ TCID_50_/mL before slightly declining. The infectious virus was also isolated from tonsil, spleen, kidney, adrenal gland, brain, colon, and mesenteric lymph node samples (Table 2), with viral titres ranging from 10^2.3^ to 10^5.5^ TCID_50_/g.

**Table 2:**
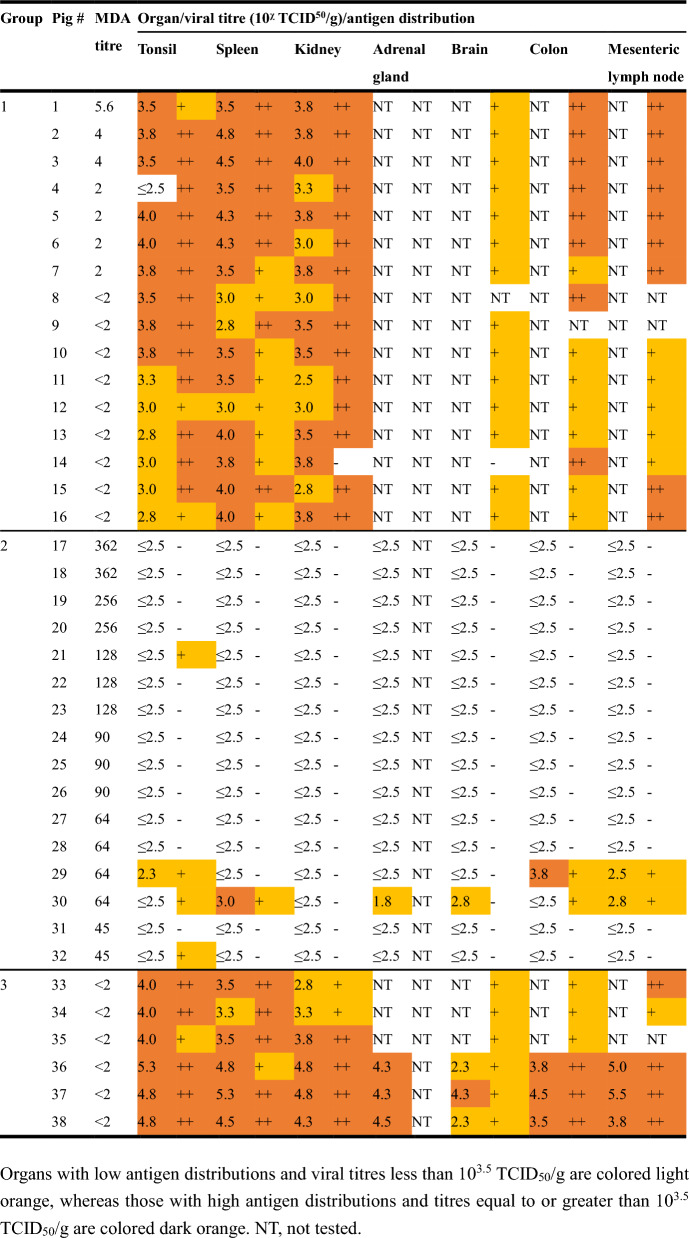
**Detection of viral antigens and titration of infectious viruses in organs collected from piglets in Groups 1 to 3**

Organs with low antigen distributions and viral titres less than 10^3.5^ TCID_50_/g are colored light orange, whereas those with high antigen distributions and titres equal to or greater than 10^3.5^ TCID_50_/g are colored dark orange. NT*,* not tested.

In Group 1, viral genes were initially detected in serum, whole blood, and oral swab samples beginning at 3 dpi (Figure 2, Additional file 4). The initial Ct values of the clinical samples were ≥ 30 for 1–4 days after the first detection and then gradually decreased to reach a minimum value of approximately 23. Viral genes were also detected in postmortem tonsil, spleen, and kidney samples, with Ct values ranging from approximately 21 to 28 (Additional file 2).

Infectious viruses were initially isolated from serum, whole blood, and oral swab samples collected from Group 1 piglets at 5, 5, and 7 dpi, respectively (Figure 3, Additional file 5). However, no infectious virus was isolated from the oral swab samples of Pig #14, which died at 9 dpi. The viral titres in the clinical samples ranged from 10^2.0^ to 10^5.1^ TCID_50_/mL and gradually increased to peak values before slightly declining. The infectious virus was also isolated from tonsil, spleen, and kidney samples, with the exception of the tonsil sample from Pig #4 (Table 2). The viral titres in the organ samples ranged from 10^2.8^ to 10^4.8^ TCID_50_/g.

In Group 2, viral genes were detected in the clinical samples collected, although the detection timing varied among individuals (Additional file 6). Compared with piglets with low MDA titres (< 128), those with high MDA titres (≥128) generally presented shorter detection periods (mean 16.4 vs 18.0 days) and fewer days with strong viral detection (Ct values < 30) (mean 0.9 vs 1.7 days), although individual variation was observed. Viral genes were detected in tonsil samples from all piglets and in varying proportions of other organs collected at necropsy or death: the spleen (10/16 piglets), kidney (4/16), adrenal gland (5/16), brain (2/16), colon (4/16), and mesenteric lymph node (12/16). The Ct values of the organ samples ranged from approximately 20 to 38 (Additional file 2).

The infectious virus was isolated only from whole blood and oral swab samples collected from Pig #30 for 2 days (11 and 13 dpi) immediately before death, with viral titres ranging from 10^2.3^ to 10^2.8^ TCID_50_/mL (Additional file 7). The virus was also isolated only from the tonsil, spleen, adrenal gland, brain, colon, and mesenteric lymph node samples of Pigs #29 and #30, with titres ranging from 10^1.8^ to 10^3.8^ TCID_50_/g (Table 2).

All groups presented 100% overall viral gene detection rates (16/16, 16/16, and 6/6 for Groups 1, 2, and 3, respectively). However, sample-specific detection rates varied among groups. The serum viral detection rates were 100% (16/16), 81.3% (13/16), and 100% (6/6) for Groups 1, 2, and 3, respectively (Group 1 vs Group 2: *p* < 0.05). The whole blood detection rates were 100%, 87.5%, and 100%, respectively (*p* > 0.05). The oral swab detection rates were 93.8%, 100%, and 100%, respectively, with no significant differences among the groups.

The infectious virus isolation rates differed dramatically among the groups. The overall isolation rates were 100% (16/16), 6.3% (1/16), and 100% (6/6) for Groups 1, 2, and 3, respectively (*p* < 0.001 for Group 2 vs Groups 1 and 3). Serum infectious virus isolation was completely prevented in Group 2 (0/16) compared with 100% in Groups 1 and 3 (*p* < 0.001). Whole blood and oral swab isolation rates were similarly lower in Group 2 (6.3% for both) than in Groups 1 and 3 (100% and 93.8–100%, respectively; *p* < 0.001).

Organ viral gene detection rates varied significantly among groups and organs. In lymphoid organs, the detection rates were as follows: tonsils 100%, 100%, and 100% for Groups 1, 2, and 3, respectively (*p* > 0.05); spleens 100%, 62.5%, and 100% for Groups 1, 2, and 3, respectively (Group 1 vs Group 2: *p* < 0.05; Group 2 vs Group 3: *p* > 0.05); and mesenteric lymph nodes 75.0% and 100% for Groups 2 and 3, respectively (*p* > 0.05). Parenchymal organ detection rates were significantly different: kidneys 100%, 25.0%, and 100% for Groups 1, 2, and 3, respectively (*p* < 0.001 for both comparisons); adrenal glands 31.3% and 100% for Groups 2 and 3, respectively (*p* > 0.05); brains 12.5 and 100% for Groups 2 and 3, respectively (*p* < 0.05); and 18.8% and 100% for Groups 2 and 3, respectively (*p* < 0.05). High MDA titres provide significant protection against viral dissemination to most organs, with stronger effects in parenchymal organs than in lymphoid organs.

Organ infectious virus isolation rates showed even more pronounced differences among the groups. In lymphoid organs, the isolation rates were as follows: tonsils 93.8%, 6.3%, and 100% for Groups 1, 2, and 3, respectively (*p* < 0.001 for all comparisons); spleens 100%, 6.3%, and 100% for Groups 1, 2, and 3, respectively (*p* < 0.001 for all Group 2 comparisons); and mesenteric lymph nodes 12.5% and 100% for Groups 2 and 3, respectively (*p* < 0.05). Parenchymal organ isolation rates demonstrated complete or near-complete protection in Group 2: kidneys 100%, 0%, and 100% for Groups 1, 2, and 3, respectively (*p* < 0.001); adrenal glands 6.3% and 100% for Groups 2 and 3, respectively (*p* < 0.01); brains 6.3% and 100% for Groups 2 and 3, respectively (*p* < 0.01); and colons 12.5% and 100% for Groups 2 and 3, respectively (*p* < 0.01). High MDA titres resulted in an 87.5–100% reduction in infectious virus isolation in organs, with complete prevention of kidney infection.

### Immunohistochemical examination

In Group 3, viral antigens were detected in all the collected organs (Table 2). Most piglets presented high viral antigen distributions in tonsil and spleen samples, whereas brain samples presented consistently low distributions.

In Group 1, viral antigens were similarly detected in all the collected organs (tonsil, spleen, kidney, brain, colon, and mesenteric lymph node) (Table 2). High antigen distribution was observed in tonsil and kidney samples from most piglets, whereas brain samples consistently presented a low distribution.

In Group 2, viral antigens were detected only in selected organs: tonsils (4/16 piglets), spleens (1/16), and colon and mesenteric lymph nodes (2/16 each) (Table 2). The remaining organs were generally negative for viral antigens.

High MDA titres provided exceptional protection against viral antigen detection. Complete or near-complete protection was observed in parenchymal organs: kidneys (93.8–100% reduction: 15/16 vs 0/16 vs 6/6 in Groups 1, 2, and 3, respectively, *p* < 0.001), brains (93.3–100% reduction: 14/15 vs 0/16 vs 3/3 in Groups 1, 2, and 3, respectively, *p* < 0.001), and colon (87.5% reduction: 15/15 vs 2/16 vs 6/6 in Groups 1, 2, and 3, respectively, *p* < 0.001). Lymphoid organs showed substantial but incomplete protection: spleen (93.8% reduction: 16/16 vs 1/16 vs 6/6 in Groups 1, 2, and 3, respectively, *p* < 0.001), mesenteric lymph nodes (87.5% reduction: 14/14 vs 2/16 in Groups 1 and 2, respectively, *p* < 0.001; 2/16 vs 5/5 in Groups 2 and 3, respectively, *p* < 0.01), and tonsils (75.0% reduction: 16/16 vs 4/16 in Groups 1 and 2, respectively, *p* < 0.001; 4/16 vs 6/6 in Groups 2 and 3, *p* < 0.01). Viral antigen breakthrough in Group 2 was limited to 4 piglets (25% of the group), predominantly those with lower MDA titres (45–128), and was restricted mainly to lymphoid organs. These findings suggested a hierarchical pattern of MDA-mediated protection: parenchymal organs > lymphoid organs.

### Antibody titres and antigenic relatedness ratios between the JPN/1/2018 and GPE^−^ strains

**Figure 4:**
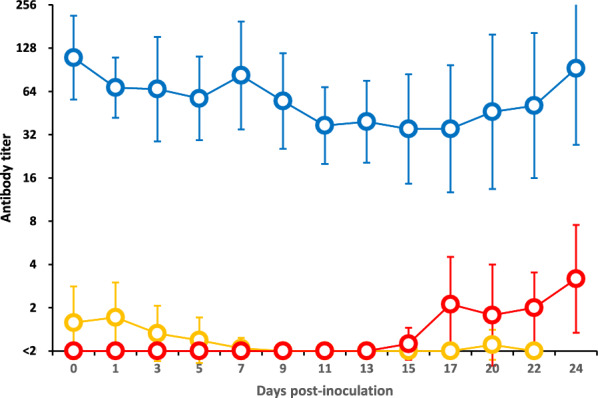
**Neutralizing antibody titres to JPN/1/2018 in piglets in Groups 1–3.** The orange, blue, and red lines represent Groups 1, 2, and 3, respectively. The error bars indicate standard deviations. In Groups 1 and 3, some piglets died during the experiment; therefore, the mean values after death were calculated using data from surviving piglets only.

In Group 3, neutralizing antibodies were undetectable from 0 to 13 dpi, but low titres ranging from 1.1 to 3.2 were detected between 15 and 24 dpi. In Group 1, low neutralizing antibody titres ranging from 1.0 to 1.7 were detected from 0 to 7 dpi, followed by minimal or undetectable levels between 9 and 22 dpi (Figure 4). In Group 2, neutralizing antibody titres gradually decreased from 109.8 to 35.1 between 0 and 17 dpi and then gradually increased from 35.1 to 92.4 between 17 and 24 dpi.

The r_1_ values between the JPN/1/2018 and GPE^−^ strains ranged from 0.18 to 1.00, with a mean r_1_ value of 0.43 (Table 3).

**Table 3:** **Antigenic relatedness ratios between the JPN/1/2018 and GPE**^−^** strains**

Group	Pig#	MDA titre against		r_1_ value
		JPN/1/2018	GPE^−^	
2	17	362	724	0.50
	18	362	512	0.71
	19	256	1024	0.25
	20	256	256	1.00
	21	128	256	0.50
	22	128	362	0.35
	23	128	362	0.35
	24	90	181	0.50
	25	90	256	0.35
	26	90	256	0.35
	27	64	362	0.18
	28	64	90	0.71
	29	64	181	0.35
	30	64	362	0.18
	31	45	181	0.25
	32	45	128	0.35
Mean		110	285	0.43

The mean values of the MDA titres are geometric mean titres, whereas the r_1_ values are arithmetic mean values.

## Discussion

This study investigated whether piglets with varying levels of MDAs acquired from GPE^−^-vaccinated sows are protected against a currently circulating virulent CSFV strain in Japan.

The MDA titres in piglets born to immunized sows decline, with a half-life of 10–14 days, primarily due to dilution effects caused by piglet growth and increased catabolism [24, 25]. Antibody test results become negative at approximately 10 weeks post-farrowing [25, 26]. Therefore, determining the minimum protective MDA titre against virulent CSFV infection and clinical manifestations is crucial for optimizing vaccination timing. Furthermore, vaccination must be administered before MDA titres fall below protective thresholds while avoiding administration too early to prevent interference with vaccine efficacy. The optimal timing may vary depending on farm-specific maternal antibody distributions. In Japan, while vaccine manufacturers recommend live vaccine administration at 30–40 days of age, maternal antibody levels on individual farms are routinely monitored by prefectural animal hygiene service centers, which then determine farm-specific vaccination schedules.

Previous studies have reported varying levels of MDA-mediated protection against CSFV. Piglets with MDAs from C strain-vaccinated sows showed age-dependent protection against lethal challenge [27], whereas those with MDAs from CP7_E2alf-vaccinated sows showed no protection against the virulent CSFV strain [6]. Similarly, 56-day-old piglets born to C strain-vaccinated sows with MDA titres of 18 ± 3 showed no protection against virulent CSFV, comparable to piglets born to rAdV-SFV-E2-vaccinated sows with 16 ± 0 MDA titres [11]. In contrast, 5-week-old piglets born to rAdV-SFV-E2-vaccinated sows with MDA titres of 49 achieved complete protection [12]. Additionally, 40–42-day-old piglets from Flc-LOM-BE^rns^-vaccinated sows with MDA titres of 64 rarely exhibited clinical manifestations, viremia or viral shedding in nasal secretions or faeces [20].

In the present study, Group 1 piglets with MDA titres ranging from < 2 to 5.6 exhibited clinical manifestations, viral loads, and viral tissue distribution patterns similar to those of the MDA-negative Group 3 piglets. In contrast, Group 2 piglets whose MDA titres ranged from 45 to 362 generally remained clinically normal, although two of the 16 piglets developed clinical signs and died during the experimental period. In these piglets, viral genes tended to be detected more frequently and at lower Ct values in animals with lower MDA titres, while infectious viruses were rarely isolated from clinical and organ samples. These results suggest that an MDA titre of 45 is the threshold for protection against clinical manifestations, infectious viremia, and infectious viral shedding caused by the JPN/1/2018 strain. This threshold aligns with previous findings where piglets with MDA titres of 49 achieved complete protection, whereas those with titres of 64 showed minimal clinical signs and viral replication [12, 20].

In contrast, two piglets in Group 2 died during the experimental period despite having MDA titres above the threshold. These piglets had smaller body sizes than others in the same group and appeared to have lower social hierarchy rankings. These findings suggest that even when piglets have MDA titres higher than the protective threshold, factors such as small body size, lower social status within the group, or underlying health conditions may compromise their protection against clinical manifestations, infectious viremia, and infectious viral shedding caused by the JPN/1/2018 strain. Taken together, these findings indicate that the MDA titre required for protection should not be considered a fixed value but rather a range that may vary depending on individual factors and physiological conditions.

A limitation of this study is the absence of piglets with MDA titres between 5.6 and 45 against the JPN/1/2018 strain, which resulted from individual variation in MDA decay rates that made precise titre prediction challenging. While this gap limits complete dose‒response characterization, the occurrence of fatal cases in Group 2 piglets with titres exceeding 45 suggests that the protective threshold lies within the evaluated range. Further studies targeting this intermediate range would refine our understanding of the MDA dose‒response relationship.

While an MDA titre in the range of 45 against the JPN/1/2018 strain appears to be protective, this threshold was determined using the JPN/1/2018 strain rather than the GPE^−^ strain. Since prefectural animal hygiene service centers in Japan use the GPE^−^ strain for neutralization tests to determine vaccination schedules on individual farms [28], it is necessary to analyse how MDA titres against the GPE^−^ strain correspond to the protective threshold titre of 45 against the JPN/1/2018 strain. The coefficient of antigenic similarity (*R* value) has traditionally been used to analyse the serological relationships between pestivirus strains, including CSFV, on the basis of antibody titres from cross-neutralization tests [29–31]. However, to understand how the MDA titre against the GPE^−^ strain corresponds to the protective threshold titre of 45 against the JPN/1/2018 strain, we applied the r_1_ value approach, which is commonly used to analyse the serological relationships between foot and mouth disease virus strains [22].

The r_1_ values between the JPN/1/2018 and GPE^−^ strains were between 0.18 and 1.00, with a mean value of 0.43. Therefore, the corresponding protective MDA titre against the GPE^−^ strain can be calculated using the following formula:

$${\text{MDA}}\;{\text{titres}}\;{\text{against}}\;{\text{the}}\;{\text{GPE - strain}} = 45 \times \frac{1}{0.43} \approx 105$$

This result suggests that piglets with MDA titres against the GPE^−^ strain below 105 should be vaccinated with the GPE^−^ strain to ensure protection against clinical manifestations, infectious viremia, and infectious viral shedding caused by the JPN/1/2018 strain. However, MDA titres against the GPE^−^ strain of ≥ 128 interfere with antibody responses to GPE^−^ strain vaccination [32]. Therefore, while an MDA titre against the GPE^−^ strain of ≥ 105 is desirable for protection against the JPN/1/2018 strain, optimal antibody acquisition requires vaccination when the MDA titre against the GPE^−^ strain is < 128. Because the MDA titres against the GPE^−^ strains 105 and 128 are very close and because the MDA titre required for protection should not be considered a fixed value but rather a range, as mentioned above, strict biosecurity measures should be implemented for piglets before vaccination to protect against clinical manifestations, infectious viremia, and infectious viral shedding caused by the JPN/1/2018 strain.

In conclusion, this study revealed that an MDA titre against the JPN/1/2018 strain of 45 represents the approximate threshold for protection against clinical manifestations, infectious viremia, and viral shedding. When converted using the antigenic relatedness value, this corresponds to an MDA titre against the GPE^−^ strain of approximately 105. However, our findings demonstrate that protection is not guaranteed by a single fixed threshold value, as individual factors such as body size, social hierarchy, and underlying health conditions may influence susceptibility even in piglets with theoretically protective antibody levels. The narrow window between the protective threshold (MDA titre against the GPE^−^ strain of 105) and the level that interferes with vaccination (MDA titre against the GPE^−^ strain of 128) presents a practical challenge for farm management.

Interestingly, survival rates did not correlate directly with MDA titre levels, with Group 3 (no MDAs) showing 100% survival despite the absence of maternal antibody protection. This finding suggests that while MDAs significantly reduced clinical manifestations, viremia, and viral shedding, the JPN/1/2018 strain exhibited moderate virulence that did not necessarily result in high mortality rates under experimental conditions.

The differential effects of MDAs on viral gene detection in different sample types provide insights into the mechanism of maternal antibody protection. High MDA titres significantly reduced viremia (serum detection) but had less pronounced effects on viral gene detection in oral swabs, suggesting that while MDAs effectively control systemic viral replication, viral RNA may still be detectable in excretions. However, the critical distinction lies in the difference between viral RNA detection and infectious virus isolation.

The dramatic reduction in infectious virus isolation rates (93.7% overall reduction) compared with viral gene detection rates reveals a critical distinction in MDA-mediated protection. While viral RNA remained detectable in many Group 2 piglets, the near-complete absence of infectious virus (particularly the 100% prevention of infectious viremia) demonstrated that high MDA titres not only suppressed viral replication but also effectively neutralized viral infectivity. This finding has profound implications for disease control, as it suggests that piglets with adequate MDA protection are unlikely to serve as sources of infection for susceptible animals, thereby contributing to herd-level immunity and reducing horizontal transmission within pig populations.

Immunohistochemical analysis revealed the most striking demonstration of MDA-mediated organ protection, with complete elimination of viral antigens in critical parenchymal organs (kidney and brain) in all high-MDA piglets. This absolute protection of vital organs represents a fundamental mechanism by which MDAs preserve essential physiological functions during CSFV challenge. The selective breakthrough in lymphoid organs (tonsil and mesenteric lymph nodes) suggests that limited viral replication at sites of initial immune surveillance may be tolerated while systemic dissemination is prevented. The restriction of antigen-positive cases to only 4 of 16 high-MDA piglets, predominantly those with lower titres within the protective range, indicates that even moderate reductions in MDA levels can compromise protection at vulnerable sites while maintaining the defense of critical organs.

Although our study included a gap in MDA titres between 5.6 and 45, which limits the precision of threshold estimation within this range, the practical relevance of our findings remains substantial for field implementation. Therefore, in addition to carefully timing vaccination on the basis of farm-specific MDA monitoring, implementing strict biosecurity measures during the vulnerable period before vaccination is essential for comprehensive protection against CSF outbreaks caused by the current strains in Japan. 

## Supplementary Information

**Additional file 1. Detection of viral genes in clinical samples collected from piglets in Group 3. **

**Additional file 2. Detection of viral genes in organs collected from piglets in Groups 1 to 3. **

**Additional file 3. Titration of infectious viruses in clinical samples collected from piglets in Group 3. **

**Additional file 4. Detection of viral genes in clinical samples collected from piglets in Group 1.**

**Additional file 5. Titration of infectious viruses in clinical samples collected from piglets in Group 1.**

**Additional file 6. Detection of viral genes in clinical samples collected from piglets in Group 2. **

**Additional file 7. Titration of infectious viruses in clinical samples collected from piglets in Group 2. **

## Data Availability

The data that support the findings of this study are available upon reasonable request from the authors.
