# Supplementary material for: The narrow window of protection: protective efficacy of maternally derived antibodies against virulent classical swine fever virus in Japan
Source: Vet Res. 2025 Jul 16;56:151. doi: 10.1186/s13567-025-01583-z (PMC12269211; doi:10.1186/s13567-025-01583-z)
Supplement: Supplementary file 1 — Additional file 1. Detection of viral genes in clinical samples collected from piglets in Group 3. [file 13567_2025_1583_MOESM1_ESM.docx]

**Additional file 1 Detection of viral genes in clinical samples collected from piglets of Group 3**

| **Pig #** | **MDA titer** | **Clinical sample** | **Days post-inoculation/Ct value** | | | | | | | | | | | | |
| --- | --- | --- | --- | --- | --- | --- | --- | --- | --- | --- | --- | --- | --- | --- | --- |
|  |  |  | **0** | **1** | **3** | **5** | **7** | **9** | **11** | **13** | **15^a^** | **17^b^** | **20** | **22** | **24** |
| 33 | <2 | S | - | - | 37.9 | 32.7 | 30.5 | 27.8 | 24.6 | 23.1 | 22.7 | 21.7 | 22.3 | NT | NT |
|  |  | WB | - | - | 37.9 | 29.0 | 28.6 | 26.0 | 22.9 | 22.1 | 21.8 | 22.0 | 21.6 | NT | NT |
|  |  | OS | - | - | 38.9 | 37.0 | 30.1 | 30.9 | 27.6 | 25.7 | 26.1 | 24.3 | 24.0 | NT | NT |
| 34 | <2 | S | - | - | 38.8 | 31.0 | 27.3 | 21.8 | 21.6 | 19.8 | 19.9 | 21.9 | 23.0 | NT | NT |
|  |  | WB | - | - | - | 30.1 | 26.6 | 20.4 | 19.6 | 18.6 | 20.5 | 21.9 | 20.5 | NT | NT |
|  |  | OS | - | - | 39.5 | 36.4 | 29.6 | 25.8 | 21.2 | 19.9 | 22.9 | 19.8 | 24.1 | NT | NT |
| 35 | <2 | S | - | - | 37.2 | 32.1 | 28.4 | 22.5 | 21.4 | 20.1 | 21.0 | 22.0 | 22.5 | NT | NT |
|  |  | WB | - | - | 36.3 | 27.4 | 26.1 | 21.1 | 19.9 | 19.8 | 20.2 | 22.3 | 20.3 | NT | NT |
|  |  | OS | - | - | - | 35.4 | 32.7 | 25.2 | 20.1 | 18.8 | 22.2 | 19.3 | 19.8 | NT | NT |
| 36 | <2 | S | - | - | 35.6 | 29.3 | 26.4 | 22.9 | 21.5 | 19.7 | 20.2 | 21.6 | 22.4 | 21.0 | NT |
|  |  | WB | - | - | 32.8 | 27.0 | 23.0 | 20.7 | 19.1 | 18.3 | 19.0 | 20.3 | 20.6 | 19.7 | NT |
|  |  | OS | - | - | - | 35.6 | 29.2 | 29.6 | 22.8 | 22.1 | 19.4 | 22.6 | 22.5 | 26.8 | NT |
| 37 | <2 | S | - | - | 34.6 | 28.2 | 24.2 | 20.9 | 19.8 | 17.6 | 16.6 | 17.7 | 18.3 | 17.6 | NT |
|  |  | WB | - | - | 31.3 | 25.7 | 22.2 | 19.4 | 18.5 | 16.2 | 17.0 | 18.9 | 18.7 | 18.3 | NT |
|  |  | OS | - | - | - | 33.9 | 29.2 | 25.2 | 22.4 | 20.7 | 15.9 | 19.9 | 17.6 | 24.2 | NT |
| 38 | <2 | S | - | - | 34.4 | 28.9 | 25.4 | 22.7 | 21.4 | 18.6 | 18.4 | 21.3 | 22.2 | 21.4 | NT |
|  |  | WB | - | 37.0 | 31.2 | 25.6 | 22.3 | 20.0 | 18.8 | 17.4 | 18.5 | 20.7 | 21.2 | 19.8 | NT |
|  |  | OS | - | - | - | 34.2 | 30.2 | 25.0 | 20.6 | 20.6 | 19.9 | 22.7 | 20.2 | 21.8 | NT |

Days with Ct values of 30 or greater are colored light orange, while those with Ct values less than 30 are colored dark orange. S, serum; WB, whole blood; OS, oral swab; NT, not tested. ^a^ The clinical samples were collected from Pigs #36 to #38 at 16 days post-inoculation. ^b^ The clinical samples were collected from Pigs #36 to #38 at 18 days post-inoculation.
