# Supplementary material for: The narrow window of protection: protective efficacy of maternally derived antibodies against virulent classical swine fever virus in Japan
Source: Vet Res. 2025 Jul 16;56:151. doi: 10.1186/s13567-025-01583-z (PMC12269211; doi:10.1186/s13567-025-01583-z)
Supplement: Supplementary file 2 — Additional file 2. Detection of viral genes in organs collected from piglets in Groups 1 to 3. [file 13567_2025_1583_MOESM2_ESM.docx]

**Additional file 2 Detection of viral genes in organs collected from piglets of Groups 1 to 3**

| **Group** | **Pig #** | **MDA titer** | **Organ/Ct value** | | | | | | |
| --- | --- | --- | --- | --- | --- | --- | --- | --- | --- |
|  |  |  | **Tonsil** | **Spleen** | **Kidney** | **Adrenal gland** | **Brain** | **Colon** | **Mesenteric lymph node** |
| 1 | 1 | 5.6 | 23.4 | 25.8 | 24.9 | NT | NT | NT | NT |
|  | 2 | 4 | 24.9 | 25.1 | 25.5 | NT | NT | NT | NT |
|  | 3 | 4 | 23.1 | 26.0 | 25.0 | NT | NT | NT | NT |
|  | 4 | 2 | 24.7 | 26.1 | 25.3 | NT | NT | NT | NT |
|  | 5 | 2 | 24.5 | 23.9 | 25.2 | NT | NT | NT | NT |
|  | 6 | 2 | 22.5 | 25.1 | 25.8 | NT | NT | NT | NT |
|  | 7 | 2 | 25.3 | 26.3 | 25.4 | NT | NT | NT | NT |
|  | 8 | <2 | 25.3 | 25.3 | 26.3 | NT | NT | NT | NT |
|  | 9 | <2 | 24.4 | 26.2 | 25.2 | NT | NT | NT | NT |
|  | 10 | <2 | 25.8 | 24.5 | 25.5 | NT | NT | NT | NT |
|  | 11 | <2 | 24.7 | 25.8 | 25.2 | NT | NT | NT | NT |
|  | 12 | <2 | 25.8 | 24.9 | 24.7 | NT | NT | NT | NT |
|  | 13 | <2 | 22.4 | 23.1 | 23.7 | NT | NT | NT | NT |
|  | 14 | <2 | 23.7 | 23.9 | 27.7 | NT | NT | NT | NT |
|  | 15 | <2 | 21.7 | 22.9 | 23.9 | NT | NT | NT | NT |
|  | 16 | <2 | 24.1 | 22.9 | 24.2 | NT | NT | NT | NT |
| 2 | 17 | 362 | 27.6 | 37.9 | - | - | - | - | 31.8 |
|  | 18 | 362 | 32.9 | - | - | - | - | - | - |
|  | 19 | 256 | 34.0 | 37.2 | - | - | - | - | - |
|  | 20 | 256 | 30.5 | 38.0 | 37.6 | - | - | - | 31.7 |
|  | 21 | 128 | 26.3 | - | - | - | - | - | 32.1 |
|  | 22 | 128 | 32.8 | - | - | - | - | - | - |
|  | 23 | 128 | 33.4 | 37.9 | - | - | - | - | 38.0 |
|  | 24 | 90 | 38.2 | - | - | - | - | - | 37.9 |
|  | 25 | 90 | 26.8 | 36.6 | - | - | - | - | 30.3 |
|  | 26 | 90 | 35.1 | 37.1 | - | 35.2 | 35.7 | - | 35.1 |
|  | 27 | 64 | 26.4 | - | - | 20.7 | - | - | 32.6 |
|  | 28 | 64 | 30.2 | - | - | - | - | - | - |
|  | 29 | 64 | 22.9 | 32.4 | 38.0 | 36.7 | - | 25.4 | 24.4 |
|  | 30 | 64 | 25.6 | 24.7 | 29.2 | 24.3 | 32.2 | 24.4 | 27.2 |
|  | 31 | 45 | 25.4 | 34.1 | - | - | - | 37.5 | 26.7 |
|  | 32 | 45 | 23.6 | 36.7 | 38.0 | 36.7 | - | 25.4 | 24.4 |
| 3 | 33 | <2 | 22.2 | 22.9 | 25.4 | NT | NT | NT | NT |
|  | 34 | <2 | 21.8 | 22.9 | 24.2 | NT | NT | NT | NT |
|  | 35 | <2 | 23.4 | 22.8 | 24.6 | NT | NT | NT | NT |
|  | 36 | <2 | 20.2 | 19.6 | 20.8 | 18.2 | 31.6 | 23.7 | 20.4 |
|  | 37 | <2 | 23.2 | 19.7 | 20.8 | 18.3 | 25.7 | 20.8 | 18.7 |
|  | 38 | <2 | 19.0 | 18.8 | 19.5 | 19.2 | 29.0 | 19.6 | 20.9 |

Organs with Ct values of 30 or greater are colored light orange, while those with Ct values less than 30 are colored dark orange. NT, not tested.
