# Supplementary material for: The narrow window of protection: protective efficacy of maternally derived antibodies against virulent classical swine fever virus in Japan
Source: Vet Res. 2025 Jul 16;56:151. doi: 10.1186/s13567-025-01583-z (PMC12269211; doi:10.1186/s13567-025-01583-z)
Supplement: Supplementary file 3 — Additional file 3. Titration of infectious viruses in clinical samples collected from piglets in Group 3. [file 13567_2025_1583_MOESM3_ESM.docx]

**Additional file 3 Titration of infectious viruses in clinical samples collected from piglets of Group 3**

| **Pig #** | **MDA titer** | **Clinical sample** | **Days post-inoculation/viral titer (10^χ^ TCID_50_/mL)** | | | | | | | | | | | | |
| --- | --- | --- | --- | --- | --- | --- | --- | --- | --- | --- | --- | --- | --- | --- | --- |
|  |  |  | **0** | **1** | **3** | **5** | **7** | **9** | **11** | **13** | **15^a^** | **17^b^** | **20** | **22** | **24** |
| 33 | <2 | S | ≤1.5 | ≤1.5 | ≤1.5 | ≤1.5 | 1.8 | 2.0 | 3.8 | 3.8 | 4.0 | 4.0 | 3.8 | NT | NT |
|  |  | WB | ≤1.5 | ≤1.5 | ≤1.5 | 2.0 | 2.3 | 3.5 | 4.3 | 3.8 | 4.5 | 4.8 | 4.0 | NT | NT |
|  |  | OS | ≤2.5 | ≤2.5 | ≤2.5 | ≤2.5 | 3.0 | 2.8 | ≤2.5 | ≤2.5 | ≤2.5 | 3.5 | 4.3 | NT | NT |
| 34 | <2 | S | ≤1.5 | ≤1.5 | ≤1.5 | 2.0 | 3.0 | 4.8 | 5.0 | 4.8 | 4.8 | 4.8 | 4.5 | NT | NT |
|  |  | WB | ≤1.5 | ≤1.5 | ≤1.5 | 3.0 | 4.3 | 4.3 | 4.8 | 5.3 | 4.8 | 4.3 | 4.8 | NT | NT |
|  |  | OS | ≤2.5 | ≤2.5 | ≤2.5 | ≤2.5 | 3.3 | 3.3 | 6.0 | 5.5 | 4.5 | 5.8 | 5.0 | NT | NT |
| 35 | <2 | S | ≤1.5 | ≤1.5 | ≤1.5 | ≤1.5 | 2.5 | 4.8 | 4.3 | 5.0 | 5.0 | 4.8 | 4.3 | NT | NT |
|  |  | WB | ≤1.5 | ≤1.5 | ≤1.5 | 2.8 | 3.0 | 4.5 | 5.0 | 5.0 | 5.3 | 5.0 | 4.8 | NT | NT |
|  |  | OS | ≤2.5 | ≤2.5 | ≤2.5 | ≤2.5 | ≤2.5 | 3.8 | 5.0 | 6.0 | 5.3 | 6.3 | 6.3 | NT | NT |
| 36 | <2 | S | ≤1.5 | ≤1.5 | ≤1.5 | ≤1.5 | 2.8 | 3.0 | 3.8 | 4.3 | 4.0 | 3.8 | 3.3 | 4.0 | 3.5 |
|  |  | WB | ≤1.5 | ≤1.5 | ≤1.5 | 2.0 | 3.5 | 4.5 | 4.3 | 4.5 | 5.3 | 4.3 | 3.8 | 4.0 | 4.0 |
|  |  | OS | ≤2.5 | ≤2.5 | ≤2.5 | ≤2.5 | ≤2.5 | 3.0 | 3.8 | 3.8 | 6.8 | 5.1 | 4.8 | 3.8 | 3.6 |
| 37 | <2 | S | ≤1.5 | ≤1.5 | ≤1.5 | ≤1.5 | 3.3 | 4.3 | 3.8 | 4.5 | 5.5 | 6.3 | 5.5 | 5.8 | 5.5 |
|  |  | WB | ≤1.5 | ≤1.5 | ≤1.5 | 1.8 | 4.0 | 4.0 | 4.0 | 4.5 | 5.5 | 5.8 | 6.0 | 6.5 | 5.8 |
|  |  | OS | ≤2.5 | ≤2.5 | ≤2.5 | ≤2.5 | 2.8 | 3.3 | 3.8 | 4.0 | 6.0 | 5.6 | 6.0 | 5.3 | 3.8 |
| 38 | <2 | S | ≤1.5 | ≤1.5 | ≤1.5 | ≤1.5 | 3.3 | 3.8 | 3.5 | 4.5 | 5.0 | 3.8 | 2.8 | 3.8 | 3.3 |
|  |  | WB | ≤1.5 | ≤1.5 | ≤1.5 | 2.8 | 3.5 | 3.5 | 3.5 | 4.8 | 4.8 | 4.5 | 4.3 | 5.0 | 5.0 |
|  |  | OS | ≤2.5 | ≤2.5 | ≤2.5 | ≤2.5 | 2.8 | 3.0 | 4.0 | 4.3 | 5.1 | 5.1 | 4.5 | 4.8 | 4.8 |

Days with viral titers less than 10^3.5^ TCID_50_/mL are colored light orange, while those with titers equal to or greater than 10^3.5^ TCID_50_/mL are colored dark orange. S, serum; WB, whole blood; OS, oral swab; NT, not tested. ^a^ The clinical samples were collected from Pigs #36 to #38 at 16 days post-inoculation. ^b^ The clinical samples were collected from Pigs #36 to #38 at 18 days post-inoculation.
