# Supplementary material for: The narrow window of protection: protective efficacy of maternally derived antibodies against virulent classical swine fever virus in Japan
Source: Vet Res. 2025 Jul 16;56:151. doi: 10.1186/s13567-025-01583-z (PMC12269211; doi:10.1186/s13567-025-01583-z)
Supplement: Supplementary file 4 — Additional file 4. Detection of viral genes in clinical samples collected from piglets in Group 1. [file 13567_2025_1583_MOESM4_ESM.docx]

**Additional file 4 Detection of viral genes in clinical samples collected from piglets of Group 1**

| **Pig #** | **MDA titer** | **Clinical sample** | **Days post-inoculation/Ct value** | | | | | | | | | | | | |
| --- | --- | --- | --- | --- | --- | --- | --- | --- | --- | --- | --- | --- | --- | --- | --- |
|  |  |  | **0** | **1** | **3** | **5** | **7** | **9** | **11** | **13** | **15** | **17** | **20** | **22** | **24** |
| 1 | 5.6 | S | - | - | 34.3 | 31.7 | 26.5 | 23.0 | 24.4 | 23.7 | 25.1 | 26.9 | 26.5 | 26.5 | NT |
|  |  | WB | - | - | 34.2 | 26.9 | 26.1 | 23.4 | 22.9 | 23.4 | 22.8 | 24.3 | 22.6 | 24.8 | NT |
|  |  | OS | - | - | 36.6 | 34.4 | 34.2 | 26.5 | 26.4 | 24.5 | 22.5 | 19.6 | 18.2 | 20.0 | NT |
| 2 | 4 | S | - | - | 34.2 | 29.9 | 25.2 | 22.3 | 23.0 | 22.7 | 22.8 | 22.5 | 24.2 | 23.2 | NT |
|  |  | WB | - | - | 33.9 | 25.5 | 27.3 | 21.1 | 24.3 | 23.2 | 22.6 | 23.6 | 24.1 | 23.0 | NT |
|  |  | OS | - | - | 39.0 | 35.1 | 28.5 | 25.5 | 27.1 | 25.6 | 25.5 | 21.6 | 19.7 | 20.9 | NT |
| 3 | 4 | S | - | - | 35.0 | 31.7 | 28.0 | 23.8 | 25.0 | 22.6 | 23.2 | 23.5 | 23.3 | 26.7 | NT |
|  |  | WB | - | - | 36.2 | 28.8 | 27.4 | 23.5 | 22.5 | 23.9 | 22.9 | 23.6 | 22.5 | 23.7 | NT |
|  |  | OS | - | - | 35.3 | 35.2 | 31.6 | 24.5 | 25.3 | 25.7 | 25.8 | 23.0 | 22.6 | 26.0 | NT |
| 4 | 2 | S | - | - | 34.4 | 29.9 | 25.3 | 23.1 | 23.5 | 22.3 | 21.4 | 22.9 | 21.3 | 24.4 | NT |
|  |  | WB | - | - | 34.6 | 26.6 | 25.4 | 22.6 | 21.8 | 24.5 | 22.5 | 23.6 | 23.2 | 23.3 | NT |
|  |  | OS | - | - | - | 35.8 | 30.4 | 23.9 | 24.6 | 24.1 | 23.8 | 21.0 | 24.6 | 19.8 | NT |
| 5 | 2 | S | - | - | 35.3 | 31.8 | 27.5 | 23.6 | 24.1 | 23.5 | 23.4 | 24.6 | 25.2 | 23.8 | NT |
|  |  | WB | - | - | 35.6 | 27.8 | 27.5 | 21.8 | 22.4 | 24.1 | 22.9 | 24.6 | 23.4 | 22.9 | NT |
|  |  | OS | - | - | 36.9 | 35.3 | 29.7 | 25.0 | 24.6 | 26.4 | 23.1 | 22.6 | 25.8 | 26.5 | NT |
| 6 | 2 | S | - | - | 36.3 | 32.2 | 28.6 | 24.7 | 24.7 | 23.8 | 22.5 | 24.5 | 27.4 | 24.2 | NT |
|  |  | WB | - | - | 35.3 | 29.2 | 26.5 | 23.5 | 23.5 | 24.6 | 22.7 | 25.4 | 24.8 | 25.9 | NT |
|  |  | OS | - | - | 35.2 | 39.5 | 33.9 | 26.6 | 24.8 | 25.6 | 24.0 | 22.4 | 23.3 | 22.3 | NT |
| 7 | 2 | S | - | - | 35.1 | 31.6 | 28.4 | 23.4 | 24.8 | 23.1 | 23.2 | 24.9 | 25.2 | 25.2 | NT |
|  |  | WB | - | - | 37.9 | 28.1 | 28.2 | 22.7 | 23.3 | 22.8 | 22.6 | 25.1 | 23.0 | 25.4 | NT |
|  |  | OS | - | - | - | - | 32.6 | 24.8 | 25.9 | 27.7 | 27.3 | 23.8 | 25.0 | 22.4 | NT |
| 8 | <2 | S | - | - | 34.4 | 31.9 | 27.0 | 24.5 | 23.8 | 22.1 | 21.3 | 24.2 | 23.3 | NT | NT |
|  |  | WB | - | - | 38.9 | 27.1 | 25.3 | 21.4 | 22.1 | 21.5 | 22.1 | 22.9 | 21.6 | NT | NT |
|  |  | OS | - | - | - | 39.9 | 30.8 | 23.9 | 22.3 | 22.9 | 24.0 | 21.7 | 19.2 | NT | NT |
| 9 | <2 | S | - | - | - | 33.1 | 27.8 | 22.9 | 22.6 | 22.2 | 22.4 | 22.5 | NT | NT | NT |
|  |  | WB | - | - | - | 28.6 | 27.1 | 21.2 | 20.6 | 24.9 | 24.0 | 22.6 | NT | NT | NT |
|  |  | OS | - | - | - | 38.3 | 31.9 | 25.8 | 25.2 | 27.0 | 25.7 | 28.0 | NT | NT | NT |
| 10 | <2 | S | - | - | 35.9 | 31.7 | 25.1 | 23.1 | 22.1 | 21.5 | 23.0 | 22.6 | 23.5 | NT | NT |
|  |  | WB | - | - | 36.9 | 26.1 | 24.0 | 21.6 | 20.9 | 21.8 | 23.8 | 22.7 | 23.3 | NT | NT |
|  |  | OS | - | - | 38.9 | 33.9 | 31.0 | 28.0 | 22.7 | 24.6 | 22.6 | 24.9 | 18.6 | NT | NT |
| 11 | <2 | S | - | - | 35.2 | 31.3 | 27.4 | 23.9 | 22.4 | 22.2 | 23.5 | 24.7 | 25.1 | NT | NT |
|  |  | WB | - | - | 35.6 | 28.6 | 28.1 | 21.9 | 21.9 | 22.4 | 23.2 | 25.3 | 22.3 | NT | NT |
|  |  | OS | - | - | - | 35.9 | 30.7 | 27.6 | 25.2 | 25.0 | 23.0 | 24.8 | 24.1 | NT | NT |
| 12 | <2 | S | - | - | 35.9 | 31.6 | 24.5 | 23.3 | 22.0 | 21.0 | 21.5 | 23.9 | 24.3 | NT | NT |
|  |  | WB | - | - | 34.8 | 25.4 | 22.8 | 20.6 | 20.6 | 21.3 | 23.0 | 24.6 | 22.8 | NT | NT |
|  |  | OS | - | - | - | 36.5 | 28.5 | 23.1 | 22.7 | 24.7 | 20.8 | 25.5 | 21.1 | NT | NT |
| 13 | <2 | S | - | - | 36.6 | 31.6 | 26.6 | 24.0 | 22.6 | 21.2 | 20.5 | 23.3 | 22.4 | NT | NT |
|  |  | WB | - | - | 35.7 | 28.2 | 26.9 | 23.9 | 23.3 | 21.5 | 22.5 | 23.2 | 20.4 | NT | NT |
|  |  | OS | - | - | 38.9 | 39.4 | 32.0 | 23.1 | 21.3 | 22.9 | 25.0 | 27.6 | 24.2 | NT | NT |
| 14 | <2 | S | - | - | 33.8 | 27.1 | 22.3 | NT | NT | NT | NT | NT | NT | NT | NT |
|  |  | WB | - | - | 34.4 | 21.0 | 20.1 | NT | NT | NT | NT | NT | NT | NT | NT |
|  |  | OS | - | - | - | 31.2 | 26.9 | NT | NT | NT | NT | NT | NT | NT | NT |
| 15 | <2 | S | - | - | 34.8 | 30.4 | 26.8 | 23.7 | 22.1 | 21.2 | 22.8 | 24.1 | 24.4 | NT | NT |
|  |  | WB | - | - | 38.0 | 27.1 | 25.7 | 22.2 | 22.6 | 21.0 | 23.5 | 24.4 | 22.0 | NT | NT |
|  |  | OS | - | - | 36.5 | 36.5 | 29.9 | 23.9 | 20.5 | 22.9 | 26.8 | 28.4 | 25.6 | NT | NT |
| 16 | <2 | S | - | - | 34.3 | 31.4 | 27.2 | 24.0 | 22.3 | 20.8 | 20.5 | 21.5 | 23.4 | NT | NT |
|  |  | WB | - | - | 36.9 | 26.6 | 25.8 | 22.4 | 21.1 | 20.6 | 21.0 | 22.7 | 20.9 | NT | NT |
|  |  | OS | - | - | 35.4 | 38.0 | 27.7 | 23.7 | 23.3 | 20.6 | 20.7 | 20.5 | 20.7 | NT | NT |

Days with Ct values of 30 or greater are colored light orange, while those with Ct values less than 30 are colored dark orange. S, serum; WB, whole blood; OS, oral swab; NT, not tested.
