# Supplementary material for: The narrow window of protection: protective efficacy of maternally derived antibodies against virulent classical swine fever virus in Japan
Source: Vet Res. 2025 Jul 16;56:151. doi: 10.1186/s13567-025-01583-z (PMC12269211; doi:10.1186/s13567-025-01583-z)
Supplement: Supplementary file 6 — Additional file 6. Detection of viral genes in clinical samples collected from piglets in Group 2. [file 13567_2025_1583_MOESM6_ESM.docx]

**Additional file 6 Detection of viral genes in clinical samples collected from piglets of Group 2**

| **Pig #** | **MDA titer** | **Clinical sample** | **Days post-inoculation/Ct value** | | | | | | | | | | | | |
| --- | --- | --- | --- | --- | --- | --- | --- | --- | --- | --- | --- | --- | --- | --- | --- |
|  |  |  | **0** | **1** | **3** | **5** | **7** | **9** | **11** | **13** | **16** | **18** | **20** | **22** | **24** |
| 17 | 362 | S | - | - | - | - | 37.5 | - | - | - | - | - | - | - | - |
|  |  | WB | - | - | - | 37.9 | 36.9 | 37.9 | - | 37.4 | - | - | - | - | - |
|  |  | OS | - | - | - | - | 38.0 | 37.9 | 36.1 | 35.4 | - | - | - | - | 38.0 |
| 18 | 362 | S | - | - | - | - | - | - | - | - | - | - | - | - | - |
|  |  | WB | - | - | - | - | - | - | - | - | - | - | - | - | - |
|  |  | OS | - | - | - | 35.9 | 29.9 | 38.1 | 38.3 | - | - | 39.9 | 37.2 | - | - |
| 19 | 256 | S | - | - | - | 36.3 | - | 37.9 | - | - | - | - | - | - | - |
|  |  | WB | - | - | 37.9 | 37.9 | 36.4 | 37.9 | - | - | - | - | - | - | - |
|  |  | OS | - | 37.4 | - | 34.0 | 38.0 | - | - | - | - | - | - | - | - |
| 20 | 256 | S | - | - | - | 36.2 | 35.8 | 36.6 | 38.0 | - | - | - | - | - | 38.1 |
|  |  | WB | - | - | - | 35.1 | 33.7 | 33.6 | 34.3 | 34.4 | - | - | - | - | - |
|  |  | OS | - | - | 36.7 | 34.4 | 31.8 | 31.2 | 35.2 | 34.6 | 37.7 | - | - | 37.6 | 37.0 |
| 21 | 128 | S | - | - | 36.0 | 33.4 | 32.9 | 33.7 | 34.6 | 34.8 | 38.0 | 37.7 | - | - | - |
|  |  | WB | - | - | 36.4 | 32.6 | 30.6 | 29.5 | 31.1 | 33.1 | 36.3 | - | - | - | - |
|  |  | OS | - | - | - | 36.3 | 35.7 | 31.0 | 33.3 | 36.0 | - | - | 38.4 | 37.9 | - |
| 22 | 128 | S | - | - | - | - | 38.0 | - | - | - | - | - | - | - | - |
|  |  | WB | - | - | - | 37.8 | 37.9 | - | 38.0 | - | 38.0 | - | - | - | - |
|  |  | OS | - | - | - | 37.7 | 28.8 | 32.3 | 33.9 | 36.7 | - | - | - | - | - |
| 23 | 128 | S | - | - | - | - | - | - | - | - | - | - | - | - | - |
|  |  | WB | - | - | - | - | 35.6 | 36.9 | 37.9 | - | - | - | - | - | - |
|  |  | OS | - | - | - | - | - | 34.2 | 37.9 | 37.7 | 38.0 | - | - | 36.2 | - |
| 24 | 90 | S | - | - | - | - | - | - | 38.1 | - | - | - | - | - | - |
|  |  | WB | - | - | - | - | 34.8 | 33.1 | 31.8 | 32.6 | 35.1 | - | - | - | - |
|  |  | OS | - | - | - | 35.9 | 32.4 | 33.5 | 30.0 | 33.6 | 33.9 | 38.3 | 36.2 | - | - |
| 25 | 90 | S | - | - | - | - | 38.0 | - | - | - | - | - | - | - | - |
|  |  | WB | - | - | 37.6 | 34.8 | 33.5 | - | 35.3 | 35.9 | 37.8 | - | - | - | - |
|  |  | OS | - | - | - | 37.8 | 34.3 | 31.5 | 35.6 | - | - | - | - | 37.8 | - |
| 26 | 90 | S | - | - | - | 34.8 | 37.4 | 37.0 | 38.0 | 36.6 | NT | NT | NT | NT | NT |
|  |  | WB | - | - | 37.0 | 35.6 | 32.4 | 32.2 | 33.8 | 33.3 | NT | NT | NT | NT | NT |
|  |  | OS | - | - | - | - | 29.7 | 28.5 | 32.2 | 33.3 | NT | NT | NT | NT | NT |
| 27 | 64 | S | - | - | - | 37.9 | - | - | - | - | - | - | - | - | - |
|  |  | WB | - | - | - | 36.3 | - | - | - | - | - | - | - | - | - |
|  |  | OS | - | 37.3 | 33.9 | 34.5 | 37.1 | 35.4 | 34.3 | 36.5 | - | - | - | - | 36.0 |
| 28 | 64 | S | - | - | 38.1 | 38.1 | 37.2 | - | - | 40.9 | - | - | - | - | - |
|  |  | WB | - | - | 36.8 | 35.0 | 33.7 | 33.6 | 34.1 | 36.2 | - | - | - | - | - |
|  |  | OS | - | - | - | - | - | 32.0 | 33.9 | - | 36.8 | - | 38.3 | 39.9 | - |
| 29 | 64 | S | - | - | - | 35.4 | 35.3 | 34.0 | 32.3 | 32.0 | 32.3 | 34.0 | 35.3 | 37.6 | 35.1 |
|  |  | WB | - | - | - | 33.3 | 30.2 | 27.8 | 27.9 | 28.5 | 30.6 | 32.8 | 34.5 | 36.8 | 36.5 |
|  |  | OS | - | - | 38.2 | - | - | 29.1 | 30.3 | 32.8 | 31.5 | 34.3 | 34.6 | 35.1 | 37.0 |
| 30 | 64 | S | - | - | - | - | 38.0 | 35.4 | 31.6 | 27.2 | NT | NT | NT | NT | NT |
|  |  | WB | - | - | - | - | 33.5 | 31.6 | 27.5 | 23.6 | NT | NT | NT | NT | NT |
|  |  | OS | - | - | - | - | 31.1 | 32.0 | 26.4 | 26.3 | NT | NT | NT | NT | NT |
| 31 | 45 | S | - | - | - | 35.7 | 34.1 | 34.0 | 33.0 | 33.1 | 32.1 | 37.1 | 38.2 | - | - |
|  |  | WB | - | - | - | 34.2 | 31.3 | 28.5 | 28.9 | 29.1 | 31.9 | 35.5 | 37.1 | - | 38.0 |
|  |  | OS | - | - | - | - | 33.9 | 29.7 | 31.5 | 35.6 | 31.3 | - | 32.6 | - | 38.0 |
| 32 | 45 | S | - | - | - | 35.8 | 34.4 | 34.9 | 34.4 | 33.8 | 38.0 | 33.9 | - | 39.7 | - |
|  |  | WB | - | - | 37.9 | 34.3 | 31.3 | 28.4 | 29.3 | 30.9 | 33.3 | 37.9 | 37.9 | - | - |
|  |  | OS | - | - | 38.1 | - | 36.2 | 29.1 | 33.8 | 31.8 | 32.8 | 36.9 | 32.5 | 35.7 | 36.4 |

Days with Ct values of 30 or greater are colored light orange, while those with Ct values less than 30 are colored dark orange. S, serum; WB, whole blood; OS, oral swab; NT, not tested.
