# Supplementary material for: The narrow window of protection: protective efficacy of maternally derived antibodies against virulent classical swine fever virus in Japan
Source: Vet Res. 2025 Jul 16;56:151. doi: 10.1186/s13567-025-01583-z (PMC12269211; doi:10.1186/s13567-025-01583-z)
Supplement: Supplementary file 7 — Additional file 7. Titration of infectious viruses in clinical samples collected from piglets in Group 2. [file 13567_2025_1583_MOESM7_ESM.docx]

**Additional file 7 Titration of infectious viruses in clinical samples collected from piglets of Group 2**

| **Pig #** | **MDA titer** | **Clinical sample** | **Days post-inoculation/viral titer (10^χ^ TCID_50_/mL)** | | | | | | | | | | | | |
| --- | --- | --- | --- | --- | --- | --- | --- | --- | --- | --- | --- | --- | --- | --- | --- |
|  |  |  | **0** | **1** | **3** | **5** | **7** | **9** | **11** | **13** | **16** | **18** | **20** | **22** | **24** |
| 17 | 362 | S | ≤1.5 | ≤1.5 | ≤1.5 | ≤1.5 | ≤1.5 | ≤1.5 | ≤1.5 | ≤1.5 | ≤1.5 | ≤1.5 | ≤1.5 | ≤1.5 | ≤1.5 |
|  |  | WB | ≤1.5 | ≤1.5 | ≤1.5 | ≤1.5 | ≤1.5 | ≤1.5 | ≤1.5 | ≤1.5 | ≤1.5 | ≤1.5 | ≤1.5 | ≤1.5 | ≤1.5 |
|  |  | OS | ≤2.5 | ≤2.5 | ≤2.5 | ≤2.5 | ≤2.5 | ≤2.5 | ≤2.5 | ≤2.5 | ≤2.5 | ≤2.5 | ≤2.5 | ≤2.5 | ≤2.5 |
| 18 | 362 | S | ≤1.5 | ≤1.5 | ≤1.5 | ≤1.5 | ≤1.5 | ≤1.5 | ≤1.5 | ≤1.5 | ≤1.5 | ≤1.5 | ≤1.5 | ≤1.5 | ≤1.5 |
|  |  | WB | ≤1.5 | ≤1.5 | ≤1.5 | ≤1.5 | ≤1.5 | ≤1.5 | ≤1.5 | ≤1.5 | ≤1.5 | ≤1.5 | ≤1.5 | ≤1.5 | ≤1.5 |
|  |  | OS | ≤2.5 | ≤2.5 | ≤2.5 | ≤2.5 | ≤2.5 | ≤2.5 | ≤2.5 | ≤2.5 | ≤2.5 | ≤2.5 | ≤2.5 | ≤2.5 | ≤2.5 |
| 19 | 256 | S | ≤1.5 | ≤1.5 | ≤1.5 | ≤1.5 | ≤1.5 | ≤1.5 | ≤1.5 | ≤1.5 | ≤1.5 | ≤1.5 | ≤1.5 | ≤1.5 | ≤1.5 |
|  |  | WB | ≤1.5 | ≤1.5 | ≤1.5 | ≤1.5 | ≤1.5 | ≤1.5 | ≤1.5 | ≤1.5 | ≤1.5 | ≤1.5 | ≤1.5 | ≤1.5 | ≤1.5 |
|  |  | OS | ≤2.5 | ≤2.5 | ≤2.5 | ≤2.5 | ≤2.5 | ≤2.5 | ≤2.5 | ≤2.5 | ≤2.5 | ≤2.5 | ≤2.5 | ≤2.5 | ≤2.5 |
| 20 | 128 | S | ≤1.5 | ≤1.5 | ≤1.5 | ≤1.5 | ≤1.5 | ≤1.5 | ≤1.5 | ≤1.5 | ≤1.5 | ≤1.5 | ≤1.5 | ≤1.5 | ≤1.5 |
|  |  | WB | ≤1.5 | ≤1.5 | ≤1.5 | ≤1.5 | ≤1.5 | ≤1.5 | ≤1.5 | ≤1.5 | ≤1.5 | ≤1.5 | ≤1.5 | ≤1.5 | ≤1.5 |
|  |  | OS | ≤2.5 | ≤2.5 | ≤2.5 | ≤2.5 | ≤2.5 | ≤2.5 | ≤2.5 | ≤2.5 | ≤2.5 | ≤2.5 | ≤2.5 | ≤2.5 | ≤2.5 |
| 21 | 128 | S | ≤1.5 | ≤1.5 | ≤1.5 | ≤1.5 | ≤1.5 | ≤1.5 | ≤1.5 | ≤1.5 | ≤1.5 | ≤1.5 | ≤1.5 | ≤1.5 | ≤1.5 |
|  |  | WB | ≤1.5 | ≤1.5 | ≤1.5 | ≤1.5 | ≤1.5 | ≤1.5 | ≤1.5 | ≤1.5 | ≤1.5 | ≤1.5 | ≤1.5 | ≤1.5 | ≤1.5 |
|  |  | OS | ≤2.5 | ≤2.5 | ≤2.5 | ≤2.5 | ≤2.5 | ≤2.5 | ≤2.5 | ≤2.5 | ≤2.5 | ≤2.5 | ≤2.5 | ≤2.5 | ≤2.5 |
| 22 | 128 | S | ≤1.5 | ≤1.5 | ≤1.5 | ≤1.5 | ≤1.5 | ≤1.5 | ≤1.5 | ≤1.5 | ≤1.5 | ≤1.5 | ≤1.5 | ≤1.5 | ≤1.5 |
|  |  | WB | ≤1.5 | ≤1.5 | ≤1.5 | ≤1.5 | ≤1.5 | ≤1.5 | ≤1.5 | ≤1.5 | ≤1.5 | ≤1.5 | ≤1.5 | ≤1.5 | ≤1.5 |
|  |  | OS | ≤2.5 | ≤2.5 | ≤2.5 | ≤2.5 | ≤2.5 | ≤2.5 | ≤2.5 | ≤2.5 | ≤2.5 | ≤2.5 | ≤2.5 | ≤2.5 | ≤2.5 |
| 23 | 128 | S | ≤1.5 | ≤1.5 | ≤1.5 | ≤1.5 | ≤1.5 | ≤1.5 | ≤1.5 | ≤1.5 | ≤1.5 | ≤1.5 | ≤1.5 | ≤1.5 | ≤1.5 |
|  |  | WB | ≤1.5 | ≤1.5 | ≤1.5 | ≤1.5 | ≤1.5 | ≤1.5 | ≤1.5 | ≤1.5 | ≤1.5 | ≤1.5 | ≤1.5 | ≤1.5 | ≤1.5 |
|  |  | OS | ≤2.5 | ≤2.5 | ≤2.5 | ≤2.5 | ≤2.5 | ≤2.5 | ≤2.5 | ≤2.5 | ≤2.5 | ≤2.5 | ≤2.5 | ≤2.5 | ≤2.5 |
| 24 | 90 | S | ≤1.5 | ≤1.5 | ≤1.5 | ≤1.5 | ≤1.5 | ≤1.5 | ≤1.5 | ≤1.5 | ≤1.5 | ≤1.5 | ≤1.5 | ≤1.5 | ≤1.5 |
|  |  | WB | ≤1.5 | ≤1.5 | ≤1.5 | ≤1.5 | ≤1.5 | ≤1.5 | ≤1.5 | ≤1.5 | ≤1.5 | ≤1.5 | ≤1.5 | ≤1.5 | ≤1.5 |
|  |  | OS | ≤2.5 | ≤2.5 | ≤2.5 | ≤2.5 | ≤2.5 | ≤2.5 | ≤2.5 | ≤2.5 | ≤2.5 | ≤2.5 | ≤2.5 | ≤2.5 | ≤2.5 |
| 25 | 90 | S | ≤1.5 | ≤1.5 | ≤1.5 | ≤1.5 | ≤1.5 | ≤1.5 | ≤1.5 | ≤1.5 | ≤1.5 | ≤1.5 | ≤1.5 | ≤1.5 | ≤1.5 |
|  |  | WB | ≤1.5 | ≤1.5 | ≤1.5 | ≤1.5 | ≤1.5 | ≤1.5 | ≤1.5 | ≤1.5 | ≤1.5 | ≤1.5 | ≤1.5 | ≤1.5 | ≤1.5 |
|  |  | OS | ≤2.5 | ≤2.5 | ≤2.5 | ≤2.5 | ≤2.5 | ≤2.5 | ≤2.5 | ≤2.5 | ≤2.5 | ≤2.5 | ≤2.5 | ≤2.5 | ≤2.5 |
| 26 | 90 | S | ≤1.5 | ≤1.5 | ≤1.5 | ≤1.5 | ≤1.5 | ≤1.5 | ≤1.5 | ≤1.5 | NT | NT | NT | NT | NT |
|  |  | WB | ≤1.5 | ≤1.5 | ≤1.5 | ≤1.5 | ≤1.5 | ≤1.5 | ≤1.5 | ≤1.5 | NT | NT | NT | NT | NT |
|  |  | OS | ≤2.5 | ≤2.5 | ≤2.5 | ≤2.5 | ≤2.5 | ≤2.5 | ≤2.5 | ≤2.5 | NT | NT | NT | NT | NT |
| 27 | 64 | S | ≤1.5 | ≤1.5 | ≤1.5 | ≤1.5 | ≤1.5 | ≤1.5 | ≤1.5 | ≤1.5 | ≤1.5 | ≤1.5 | ≤1.5 | ≤1.5 | ≤1.5 |
|  |  | WB | ≤1.5 | ≤1.5 | ≤1.5 | ≤1.5 | ≤1.5 | ≤1.5 | ≤1.5 | ≤1.5 | ≤1.5 | ≤1.5 | ≤1.5 | ≤1.5 | ≤1.5 |
|  |  | OS | ≤2.5 | ≤2.5 | ≤2.5 | ≤2.5 | ≤2.5 | ≤2.5 | ≤2.5 | ≤2.5 | ≤2.5 | ≤2.5 | ≤2.5 | ≤2.5 | ≤2.5 |
| 28 | 64 | S | ≤1.5 | ≤1.5 | ≤1.5 | ≤1.5 | ≤1.5 | ≤1.5 | ≤1.5 | ≤1.5 | ≤1.5 | ≤1.5 | ≤1.5 | ≤1.5 | ≤1.5 |
|  |  | WB | ≤1.5 | ≤1.5 | ≤1.5 | ≤1.5 | ≤1.5 | ≤1.5 | ≤1.5 | ≤1.5 | ≤1.5 | ≤1.5 | ≤1.5 | ≤1.5 | ≤1.5 |
|  |  | OS | ≤2.5 | ≤2.5 | ≤2.5 | ≤2.5 | ≤2.5 | ≤2.5 | ≤2.5 | ≤2.5 | ≤2.5 | ≤2.5 | ≤2.5 | ≤2.5 | ≤2.5 |
| 29 | 64 | S | ≤1.5 | ≤1.5 | ≤1.5 | ≤1.5 | ≤1.5 | ≤1.5 | ≤1.5 | ≤1.5 | ≤1.5 | ≤1.5 | ≤1.5 | ≤1.5 | ≤1.5 |
|  |  | WB | ≤1.5 | ≤1.5 | ≤1.5 | ≤1.5 | ≤1.5 | ≤1.5 | ≤1.5 | ≤1.5 | ≤1.5 | ≤1.5 | ≤1.5 | ≤1.5 | ≤1.5 |
|  |  | OS | ≤2.5 | ≤2.5 | ≤2.5 | ≤2.5 | ≤2.5 | ≤2.5 | ≤2.5 | ≤2.5 | ≤2.5 | ≤2.5 | ≤2.5 | ≤2.5 | ≤2.5 |
| 30 | 64 | S | ≤1.5 | ≤1.5 | ≤1.5 | ≤1.5 | ≤1.5 | ≤1.5 | ≤1.5 | ≤1.5 | NT | NT | NT | NT | NT |
|  |  | WB | ≤1.5 | ≤1.5 | ≤1.5 | ≤1.5 | ≤1.5 | ≤1.5 | 2.3 | 2.8 | NT | NT | NT | NT | NT |
|  |  | OS | ≤2.5 | ≤2.5 | ≤2.5 | ≤2.5 | ≤2.5 | ≤2.5 | 2.8 | 2.8 | NT | NT | NT | NT | NT |
| 31 | 45 | S | ≤1.5 | ≤1.5 | ≤1.5 | ≤1.5 | ≤1.5 | ≤1.5 | ≤1.5 | ≤1.5 | ≤1.5 | ≤1.5 | ≤1.5 | ≤1.5 | ≤1.5 |
|  |  | WB | ≤1.5 | ≤1.5 | ≤1.5 | ≤1.5 | ≤1.5 | ≤1.5 | ≤1.5 | ≤1.5 | ≤1.5 | ≤1.5 | ≤1.5 | ≤1.5 | ≤1.5 |
|  |  | OS | ≤2.5 | ≤2.5 | ≤2.5 | ≤2.5 | ≤2.5 | ≤2.5 | ≤2.5 | ≤2.5 | ≤2.5 | ≤2.5 | ≤2.5 | ≤2.5 | ≤2.5 |
| 32 | 45 | S | ≤1.5 | ≤1.5 | ≤1.5 | ≤1.5 | ≤1.5 | ≤1.5 | ≤1.5 | ≤1.5 | ≤1.5 | ≤1.5 | ≤1.5 | ≤1.5 | ≤1.5 |
|  |  | WB | ≤1.5 | ≤1.5 | ≤1.5 | ≤1.5 | ≤1.5 | ≤1.5 | ≤1.5 | ≤1.5 | ≤1.5 | ≤1.5 | ≤1.5 | ≤1.5 | ≤1.5 |
|  |  | OS | ≤2.5 | ≤2.5 | ≤2.5 | ≤2.5 | ≤2.5 | ≤2.5 | ≤2.5 | ≤2.5 | ≤2.5 | ≤2.5 | ≤2.5 | ≤2.5 | ≤2.5 |

Days with viral titers less than 10^3.5^ TCID_50_/mL are colored light orange. S, serum; WB, whole blood; OS, oral swab; NT, not tested.
